# Preparation of ZnSO_4_·7H_2_O using filter cake enriched in calcium and magnesium from the process of zinc hydrometallurgy

**DOI:** 10.1038/s41598-017-16575-z

**Published:** 2017-11-24

**Authors:** Bo Li, Xuanbing Wang, Yonggang Wei, Hua Wang, Guanglai Hu

**Affiliations:** 1 State Key Laboratory of Complex Nonferrous Metal Resources Clean Utilization, Kunming University of Science and Technology, Kunming, 650093 China; 2 Faculty of Metallurgy and Energy Engineering, Kunming University of Science and Technology, Kunming, 650093 China

## Abstract

ZnSO_4_∙7H_2_O was prepared using a filter cake enriched in calcium and magnesium that was generated during the process of zinc hydrometallurgy. The study was optimized to obtain process parameters. The results show that the optimal acid leaching parameters are a solid-to-liquid ratio of 1:3.5, a sulfuric acid concentration of 16%, an acid leaching time of 20 min, a final pH of 4.1–4.4, a cooling and settling time of 120 min, an oxidation time of 20 min, a stirring speed of 300 r/min, a H_2_O_2_ dosage of 25 mL/L, a crystallization temperature of 20 °C, and a crystallization time of 60 min. The ZnSO_4_∙7H_2_O content in the product is 98.6%, and the zinc recovery efficiency is 97.5%. This process is characterized by simple flow and low cost, while the circulation and accumulation problems with calcium and magnesium ions in the zinc hydrometallurgy process are also solved.

## Introduction

During the hydrometallurgical process of zinc, calcium and magnesium enter into the system with the raw materials in the form of sulfate and thus cannot be removed like Cu, Co, Cd, etc^1^., which are generally removed by cementation onto zinc. Calcium and magnesium ions continue to accumulate until the solution is saturated. This results in a number of challenges, including a large quantity of calcium and magnesium salts produced in the zinc plant. The presence of these salts increases the viscosity of the solution and makes solid-liquid separation more difficult. When the concentrations of calcium and magnesium ions come close to saturation by partial temperature reduction, they precipitate mainly in the form of sulfate or hydrated sulfate. This damages equipment and causes blockage of pipes during the subsequent extraction process. Furthermore, in the zinc electrowinning process, a high calcium and magnesium ion content increases resistance, thus reducing current efficiency^2^. To solve these problems, cooling fans and settling tanks are used to remove calcium and magnesium deposits. However, this operation gives rise to another problem: how to treat the filter cake enriched in calcium and magnesium.

**Figure 1:**
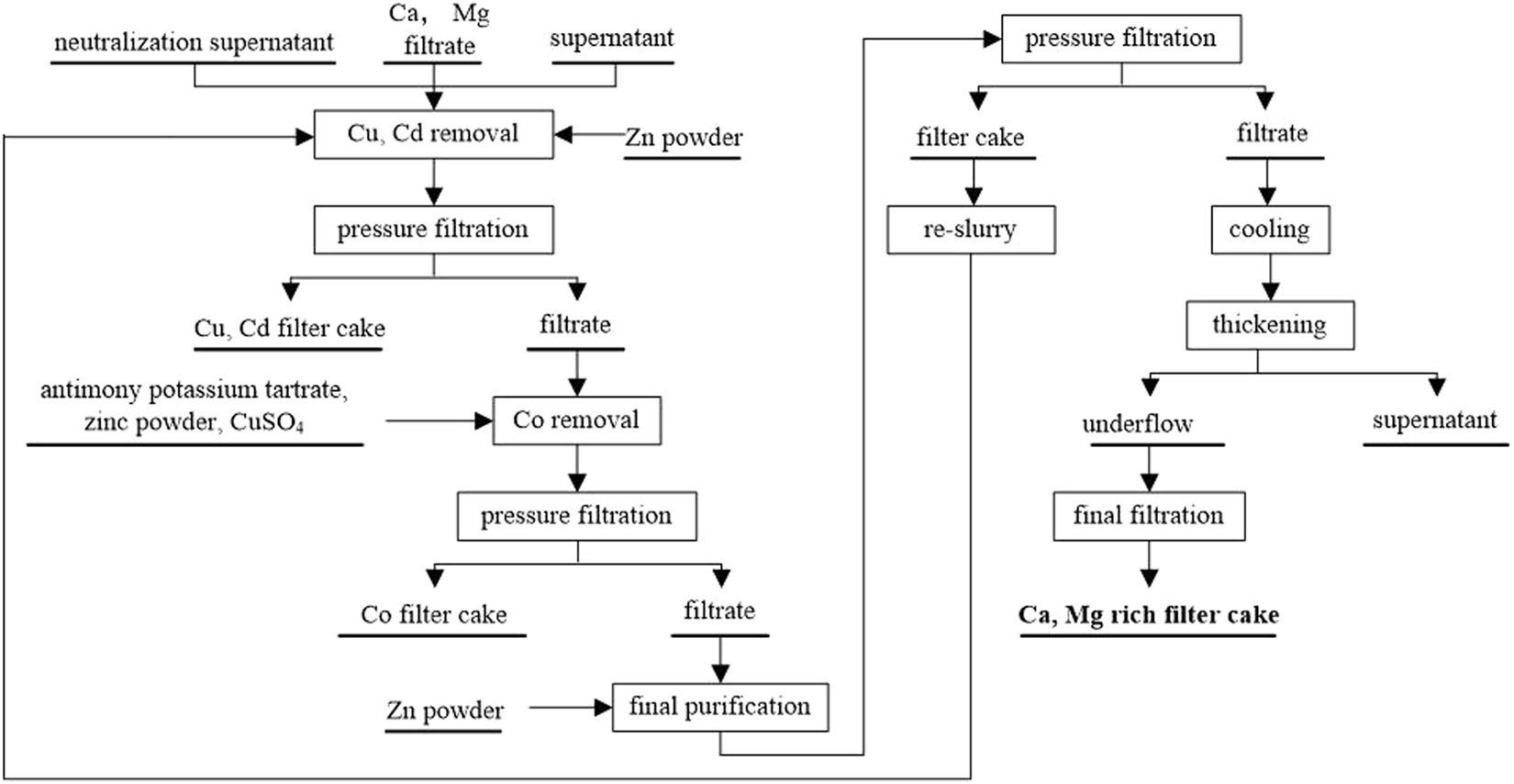
The flow sheet of three-stage purification of zinc hydrometallurgy.

The general flow of the three-stage purification process is shown in Fig. 1. In the flow sheet, Cu and Cd are removed in the first stage with the reaction Zn + Me^2+^ = Me + Zn^2+^ (Me = Cu and Cd). At the second purification stage, Co and Ni are removed as well, but the Cd will re-dissolute into the solution from the precipitate. The concentration of Cd^2+^ must be kept in an extremely low range, so the third operation is added to remove Cd^2+^. During whole operations, three factors must be severely controlled in a suitable range, including temperature, the weight of zinc and agitation speed. Ca and Mg cannot be removed in this whole operation, so the underflow generated at the end of the process enriched in zinc, calcium and magnesium. Therefore, producing ZnSO_4_·7H_2_O using a filter cake enriched in calcium and magnesium is an excellent solution. Globally, 30% zinc is produced from zinc concentrate, and the annual renewable zinc output can be up to 2.9 million tons. However, in China, regenerated zinc accounts are far short of the global level, at only approximately 13% of the total zinc output^3^. Preparation of ZnSO_4_∙7H_2_O using the filter cake enriched in calcium and magnesium is an appropriate strategy to conserve energy and to reduce emissions^4^.

High-quality ZnSO_4_∙7H_2_O is an important industrial raw material that has a wide range of applications in medicine, electroplating, the industrial production of artificial fibers, pesticides, chemical reagents, and more^5^. Recently, preparing ZnSO_4_∙7H_2_O from different secondary resources has become a research focal point in non-ferrous metallurgy due to its increasing global demand. The filter cake is rich in calcium and magnesium and contains a high proportion of zinc, which is a favorable crude material for the preparation of ZnSO_4_∙7H_2_O.

Early in the 1980s, researchers prepared ZnSO_4_∙7H_2_O using industrial waste residue and wastewater from a zinc hydrometallurgical processing plant. These processes still suffer from low recovery efficiency as well as low and unstable product quality. In recent years, due to increasing zinc prices and to reduce enterprise production costs, some researchers are exploring the use of industrial wastewater and waste residue to produce ZnSO_4_∙7H_2_O. Chinese researchers have used wastewater from zinc hydrometallurgy to produce ZnSO_4_∙7H_2_O via washing, purification, concentration, and crystallization. This approach offers considerable economic benefits. The ZnSO_4_∙7H_2_O is prepared from smithsonite (ZnCO_3_) by a process involving roasting, leaching, impurity removal, evaporating, cooling, and crystallization. Sphalerite (ZnS) can also be used after calcination at 750 °C by reacting the calcine with sulfuric acid. The effects of calcination temperature, sulfuric acid concentration, reaction time, and reaction temperature on the leaching efficiency were studied to optimize the experimental conditions^6–11^.

Some researchers have prepared ZnSO_4_∙7H_2_O using leaching, solvent extraction with P204 (di-(2-ethylhexyl) phosphoric acid), stripping, concentrating, and separating using crystallization. The results showed that with P204 as the solvent and with the use of a neutralizing agent, the extraction efficiency can reach more than 99%. Ion impurities remained in the raffinate, and the Zn^2+^ in the procedure had a low loss efficiency at the same time. Sulfuric acid was used to strip the loaded organic phase, which produced a solution of high zinc concentration. This method separated impurities and improved the recovery efficiency of zinc, resulting in a solution with a high concentration of zinc and a low amount of impurities^12–15^.

In this paper, ZnSO_4_∙7H_2_O is produced from a filter cake enriched in calcium and magnesium. This method stands out for its simple production route, affordable cost and high profit margin. In addition, it solves the problem of properly settling this thicker sludge.

## Test Materials and Methods

### Test materials

The filter cake enriched in calcium and magnesium utilized in this study was obtained from Yunnan Chihong Zn&Ge Co., Ltd., China. The filter cake samples were crushed, and a size analysis was performed by sieving, which indicated that the fine fraction (150–180 μm) constituted 90 wt% of the samples.

### Test apparatus and method

Samples were characterized using a Japan Science X-ray diffractometer with Cu Kα radiation (λ = 1.5406 Å), an operating voltage of 40 kV, and a current of 40 mA. The diffraction angle (2*θ*) was scanned from 10 to 90 deg. Scanning electron microscopy (SEM; HITACHI-S3400N) was also performed. In the leaching, removal, and crystallization processes, the concentrations of Zn^2+^, Fe^3+^ and Ca^2+^ were analyzed using titrimetry with EDTA(ethylene diamine tetraacetic acid). The content of ZnSO_4_∙7H_2_O in the product was analyzed by chemical analysis.

**Figure 2:**
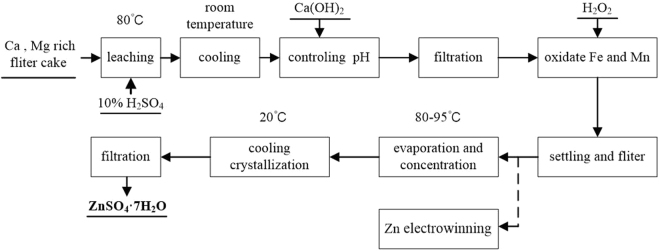
The general process flow sheet of prepare of ZnSO_4_∙7H_2_O using the filter cake of enriched calcium and magnesium from the process of zinc hydrometallurgy.

The general process flow sheet for the production of ZnSO_4_∙7H_2_O from a pretreatment filter cake enriched in calcium and magnesium (Fig. 2) involves six steps: acid leaching, cooling to remove Ca^2+^ and Mg^2+^, preliminary removal of Fe^3+^, removal of oxidized Fe and Mn, evaporation and concentration, crystallization, and drying. The output waste filter cake is sent back to the rotary kiln^16^. The supernatant, which is obtained by settling and filtering, may also be processed directly in the zinc electrowinning process.

### Acid leaching

Acid leaching tests were performed in a 300-mL beaker with the temperature controlled by a water bath at 80 °C. The 16 wt% H_2_SO_4_ solution and filter cake samples were prepared according to a specific solid-liquid ratio. They were homogeneously mixed in a beaker using an electric blender at a stirring speed of 200 rpm for 20 min, followed by 10 min of settling. After the reaction, the liquid-solid slurry was separated by pump filtration. The filtrate was rich in soluble Zn and Ca, and the filter cake was washed, dried, and then sampled for analysis.

### Cooling, settling and removal of iron and calcium

The tests for cooling and settling were performed in a 300-mL beaker. The solution was allowed to cool slowly in air, and a lime emulsion was added to adjust the pH during the process. The pH of the solution was measured using a pH meter. This was followed by a settling period. Finally, the precipitate obtained at the bottom of the beaker was washed, dried, and sampled for analysis.

### Oxidative removal of iron and manganese

The oxidation was carried out by the addition of hydrogen peroxide (H_2_O_2_). The slurry was stirred at different stirring speeds over different time periods. Finally, the precipitate obtained at the bottom of the beaker was filtered, washed, dried, and sampled for analysis.

### Concentration by evaporation and crystallization by cooling

The purified solution was concentrated at a temperature range from 80 °C to 95 °C, and the temperature was controlled by a water bath at 60 °C until saturation and crystallization of the zinc sulfate. The solution was filtered to collect the sample.

## Discussion

### Physical and chemical characteristics of the material

**Figure 3:**
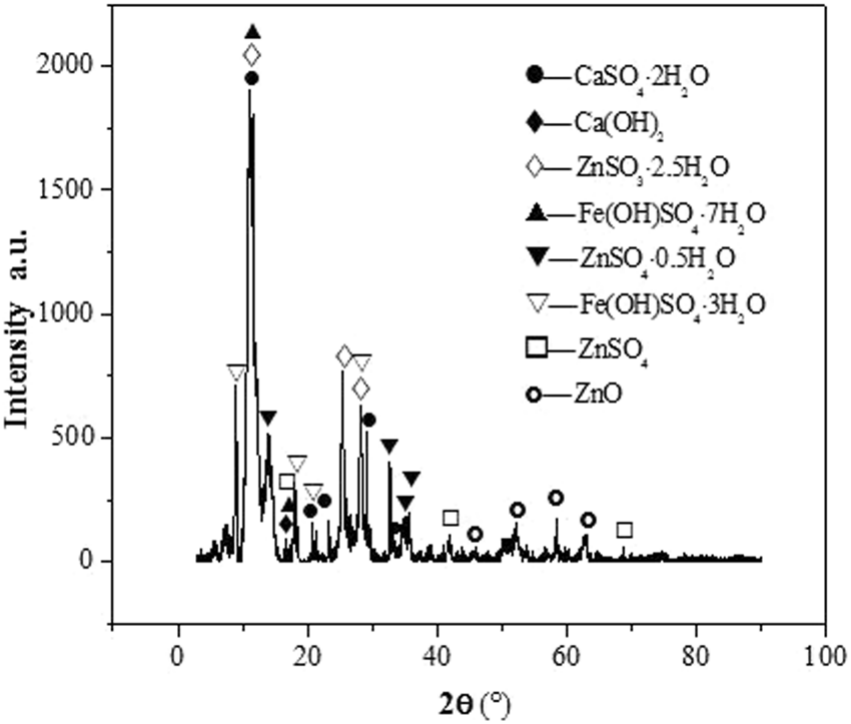
XRD pattern of the filter cake enrich in calcium and magnesium.

**Figure 4:**
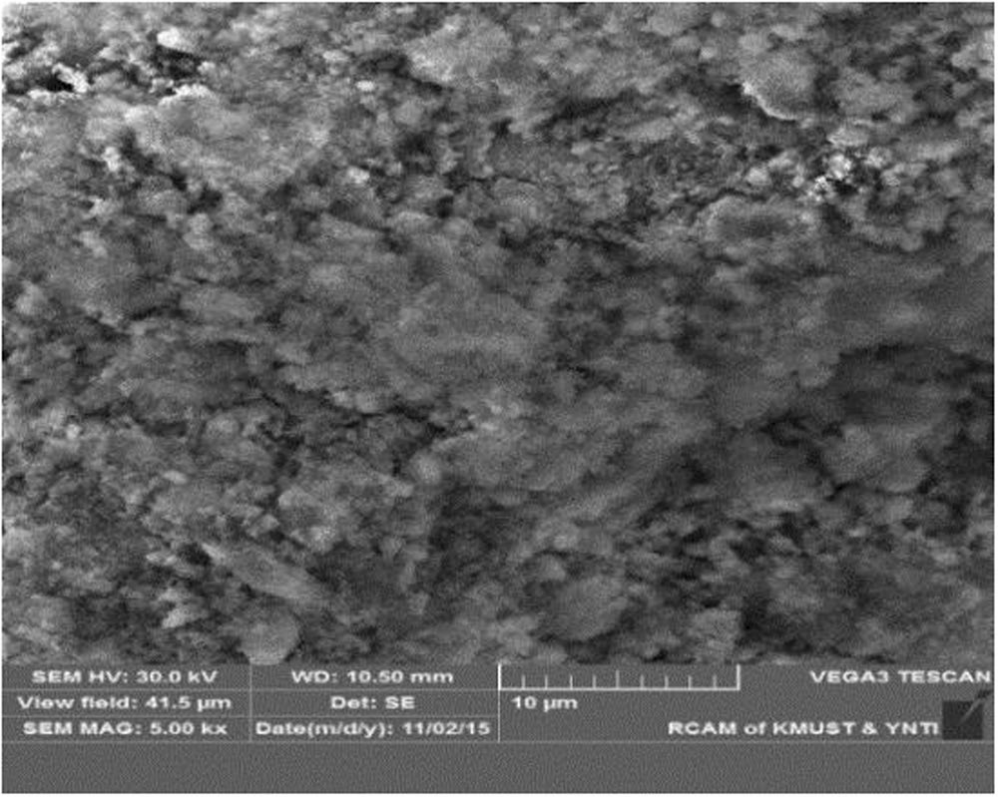
SEM image of the filter cake rich in calcium and magnesium.

The filter cake consists of white powder and a certain amount of water. The sample was mainly composed of zinc, oxygen, and sulfur, with smaller amounts of iron, calcium, magnesium, and manganese and traces of copper, cadmium, and arsenic (Table 1). The XRD analysis in Fig. 3 shows that the filter cake is mainly composed of ZnSO_4_∙nH_2_O, Fe(OH)_3_, MgSO_4_∙nH_2_O, CaSO_4_∙nH_2_O, and Ca(OH)_2_. The SEM result in Fig. 4 shows the presence of flocculent pieces in the samples.

**Table 1:** Chemical composition of filter cake rich in calcium and magnesium.

Element	Zn	O	S	Fe	Ca	Mn	Mg
Content (wt. %)	48.93	35.02	7.351	0.151	1.880	0.197	0.113

### Sulfuric acid leaching

The leaching process occurs according to the following chemical reactions^17–19^.

1 $${\rm{ZnO}}+{{\rm{H}}}_{2}{{\rm{SO}}}_{4}={{\rm{ZnSO}}}_{4}+{{\rm{H}}}_{2}{\rm{O}}$$

2 $${{\rm{ZnSO}}}_{4}\cdot {{\rm{nH}}}_{2}{\rm{O}}={{\rm{ZnSO}}}_{4}+{{\rm{nH}}}_{2}{\rm{O}}$$

3 $$2{\rm{Fe}}({\rm{OH}}){{\rm{SO}}}_{4}\cdot {{\rm{nH}}}_{2}{\rm{O}}+{{\rm{H}}}_{2}{{\rm{SO}}}_{4}={{\rm{Fe}}}_{2}{({{\rm{SO}}}_{4})}_{3}+(n+2){{\rm{H}}}_{2}{\rm{O}}$$

4 $${\rm{Ca}}{({\rm{OH}})}_{2}+{{\rm{H}}}_{2}{{\rm{SO}}}_{4}={{\rm{CaSO}}}_{4}+2{{\rm{H}}}_{2}{\rm{O}}$$

The effect of H_2_SO_4_ concentration (10 wt% to 22 wt%) on the leaching of Zn from the filter cake of enriched calcium and magnesium at a leaching temperature of 80 °C, solid-liquid ratio 1:3.0 (g/mL), leaching time of 20 min and stirring speed of 200 rpm is summarized in Table 2. The amount of Zn leached increased considerably with the increase in the H_2_SO_4_ concentration from 10 wt% to 22 wt%. This result indicates that the H_2_SO_4_ solution concentration has a significant effect on the leaching of Zn, and the optimal H_2_SO_4_ solution concentration is 16 wt%.

**Table 2:** Effect of H_2_SO_4_ solution concentration on leaching efficiency.

H_2_SO_4_ (wt%)	Leaching residue mass (g)	Zn content in residue (wt%)	Zn concentration in solution (g/L)	Leaching efficiency (%)
10	7.96	10.32	154	94.4
13	4.71	7.18	159	97.7
16	1.08	5.83	162	99.6
19	1.01	4.96	163	99.7
22	1.07	4.51	163	99.7

The effect of changing the solid-liquid ratio at an H_2_SO_4_ solution concentration of 16 wt%, stirring speed of 200 rpm, and leaching temperature of 80 °C was investigated, and the results are listed in Table 3. The amount of leached Zn increased as the solid-liquid ratio increased from 1:3.0–1:5.0 (g/mL), and the leached Zn leveled off beyond this solid-liquid ratio. The optimal solid-liquid ratio for leaching is 1:3.5.

**Table 3:** Effect of solid-liquid ratio on leaching efficiency.

Solid-liquid ratio (g/mL)	Leaching residue mass (g)	Zn content in residue (wt%)	Zn concentration in solution (g/L)	Leaching efficiency (%)
1: 3.0	3.20	11.25	163	97.5
1: 3.5	0.82	5.63	140	99.7
1: 4.0	0.74	5.00	122	99.7
1: 4.5	0.68	3.75	109	99.8
1: 5.0	0.45	4.06	97.9	99.9

The effect of leaching time was investigated in the range of 10 to 150 min at a H_2_SO_4_ solution concentration of 16 wt%, leaching temperature of 80 °C, and solid-liquid ratio of 1:3.5, and the results are shown in Table 4. The amount of Zn leached increased rapidly in the initial stages of the leaching process and leveled off after 20 min. The maximum amount of Zn leached reached 99.9 wt%. This indicates that 20 min is sufficient for maximum Zn leaching efficiency. Since the efficiency of the reaction is controlled by diffusion through the solid layer around the shrinking unreacted core, then the efficiency increases with the growth of time during a suitable range. It was found that the efficiency levels off beyond a certain duration^20^.

**Table 4:** Effect of leaching time on leaching efficiency.

Time (min)	Leaching residue mass (g)	Zn content in residue (wt%)	Zn concentration in solution (g/L)	Leaching efficiency (%)
10	2.93	6.81	161	98.6
20	1.25	7.02	162	99.6
30	0.79	4.38	163	99.8
60	0.77	2.50	163	99.9
90	0.82	3.75	163	99.8
120	0.97	3.13	163	99.8
150	0.80	2.50	163	99.8

### Cooling, settling, and removal

The effect of the final pH on the removal of Ca and Fe from a 16 wt% H_2_SO_4_ solution is shown in Table 5. The removal of Fe increased considerably with an increase in pH, leveling off after a certain value was reached. Removal of Ca increased slowly with an increase in the final pH and began to decrease when the pH exceeded 4.4. The final pH also affected the recovery of zinc. In summary, the optimal final pH is 4.1–4.4. This range offers low Mg^2+^ concentrations with high solubility. Therefore, it was concluded that the removal of Mg^2+^ occurs via crystallization. The Mg^2+^ stays in the mother liquid, which is returned to the accumulation process when the Mg^2+^ concentration reaches a certain level via a two-stage cooling subsidence removal process. The Mg^2+^ content was maintained at a certain level. CaSO_4_ and Fe(OH)_3_ in the solution underwent the following main chemical reactions during the removal process.

5 $${{\rm{Ca}}}^{2+}({\rm{aq}})+{{\rm{SO}}}_{4}^{2-}\,({\rm{aq}})={{\rm{CaSO}}}_{4}({\rm{s}})$$

6 $${{\rm{Fe}}}^{3+}+3{{\rm{H}}}_{2}{\rm{O}}={\rm{Fe}}{({\rm{OH}})}_{3}+3{{\rm{OH}}}^{-}$$

**Table 5:** Effect of end point pH on precipitation efficiency.

pH	Residue mass (g)	Fe mass in residue (g)	Ca removed mass (g)	Fe precipitation efficiency (%)	Ca precipitation efficiency (%)
3.0–3.8	1.62	0.011	0.140	24.3	24.8
3.8–4.1	2.66	0.024	0.253	53.0	44.9
4.1–4.4	2.69	0.027	0.265	59.6	47.0
4.4–4.6	2.35	0.029	0.184	64.0	32.6
4.6–4.8	2.33	0.029	0.171	62.9	30.3
4.8–5.4	2.30	0.029	0.156	64.0	27.7

The solubility product of CaSO_4_ is 3.16 × 10^–7^, and the SO_4_^2−^ concentration is 1.83 mol/L. When a lime emulsion was added into the solution, CaSO_4_ precipitated at the bottom of the beaker. The precipitation efficiency decreased with the increase in lime emulsion, and excess Ca^2+^ remained in the solution. At a pH of 4.1–4.4, Fe^3+^ precipitated as Fe(OH)_3_ (_S_) (equation 6). There was no change in the precipitation efficiency of Fe^3+^ if the pH was increased beyond 4.4, but a significant amount of Zn^2+^ was adsorbed and carried into the precipitation. Thus, there was no observed advantage to using a pH higher than 4.2.

The precipitation efficiencies of Fe, Ca, Mg, and other impurities increased as the settlement time increased. The impurities in the filter cake phase were removed. Table 6 shows experimentally derived settling times. The precipitation efficiency of Ca and Fe leveled off at 120 min.

**Table 6:** Effect of settling time on precipitation efficiency.

Time/min	Residue mass (g)	Fe mass in residue (g)	Ca removed mass (g)	Fe precipitation efficiency (%)	Ca precipitation efficiency (%)
30	1.59	0.019	0.108	41.9	19.2
60	1.86	0.023	0.139	50.2	24.6
90	2.10	0.025	0.174	55.2	30.8
120	2.61	0.030	0.278	66.2	49.3
150	2.91	0.032	0.270	70.6	47.9

### Oxidative removal of iron

**Figure 5:**
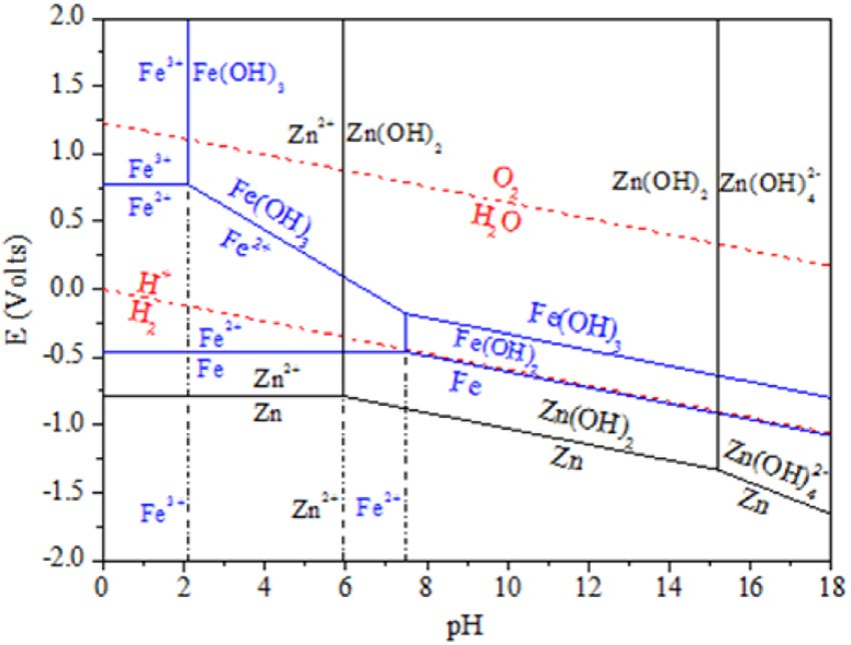
E-pH diagram of the Zn-Fe-H_2_O system at 25 °C.

**Figure 6:**
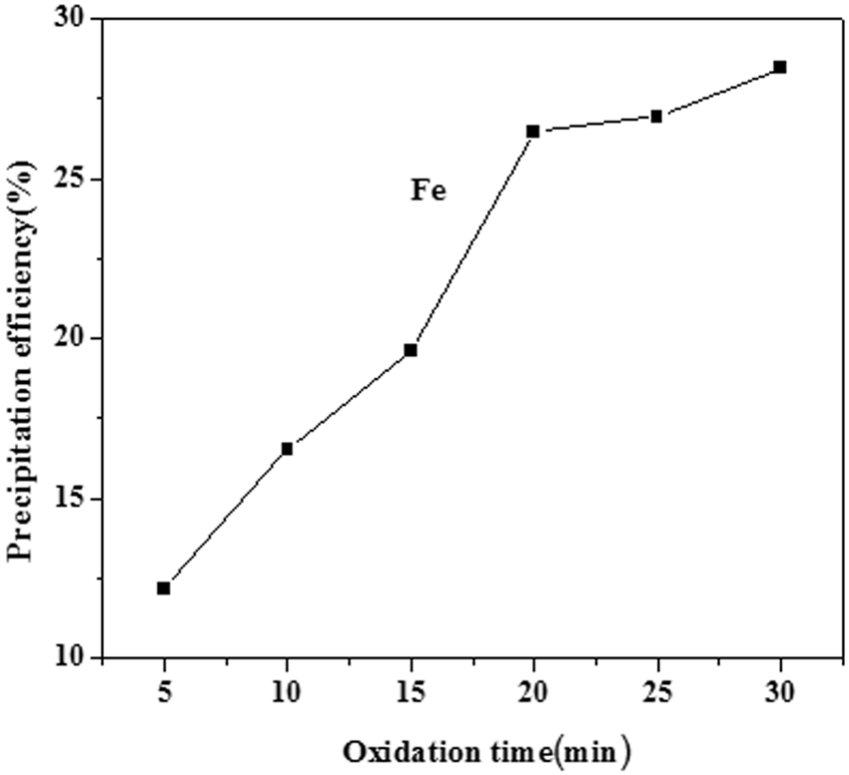
Effect of oxidation time.

Figure 5 shows the E–pH diagram of the Zn–Fe–H_2_O systems at 25 °C. The order of the pH values of the three ions at initial precipitation was Fe^3+^ < Zn^2+^ < Fe^2+^. When the Zn^2+^ started to hydrolyze, the Fe^3+^ had been hydrolyzed to a greater extent, while the Fe^2+^ had not yet hydrolyzed. Therefore, the Fe^3+^ ions can be removed by Fe(OH)_3_ formation and precipitation. However, Fe^2+^ cannot be removed by means of a neutralizing acid directly, so Fe^2+^ must be oxidized to Fe^3+^ by an added oxidative reagent. The effect of the 5 to 30 min oxidation time on the removal of Fe was investigated, and the results are shown in Fig. 6. In this process, ferrous ions were oxidized to ferric ions, and the balanced chemical reaction is shown in equation (7). The removal efficiency of Fe leaching increased considerably with an increasing oxidation time from 5 to 30 min, after which the amount of Fe leached levels off. The Fe^2+^ ions can be removed by conversion to Fe(OH)_3_ using hydrogen peroxide (H_2_O_2_), and Mn^2+^ can be removed by conversion to MnO_2_. The precipitation of Fe(OH)_3_ in the colloidal form is a slow process. The colloidal particles grow over time^21,22^. However, these particles may incorporate zinc as their particle diameters become larger. In summary, the optimal oxidation time is 20 min.

7 $$2{{\rm{FeSO}}}_{4}+{{\rm{H}}}_{2}{{\rm{O}}}_{2}+{{\rm{H}}}_{2}{{\rm{SO}}}_{4}={{\rm{Fe}}}_{2}{({{\rm{SO}}}_{4})}_{3}+2{{\rm{H}}}_{2}{\rm{O}}$$

**Figure 7:**
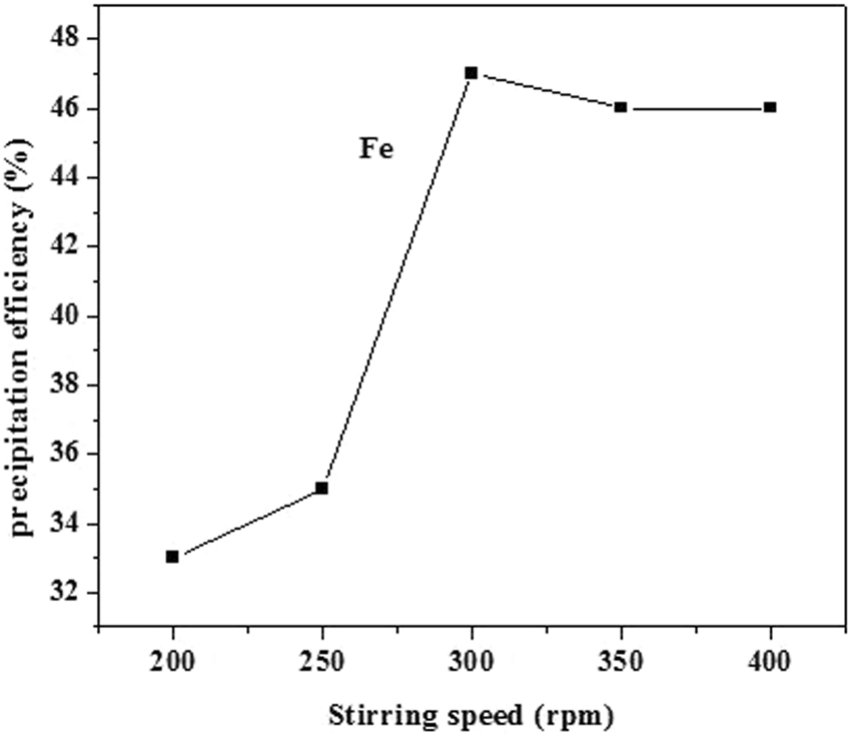
Effect of stirring speed.

**Figure 8:**
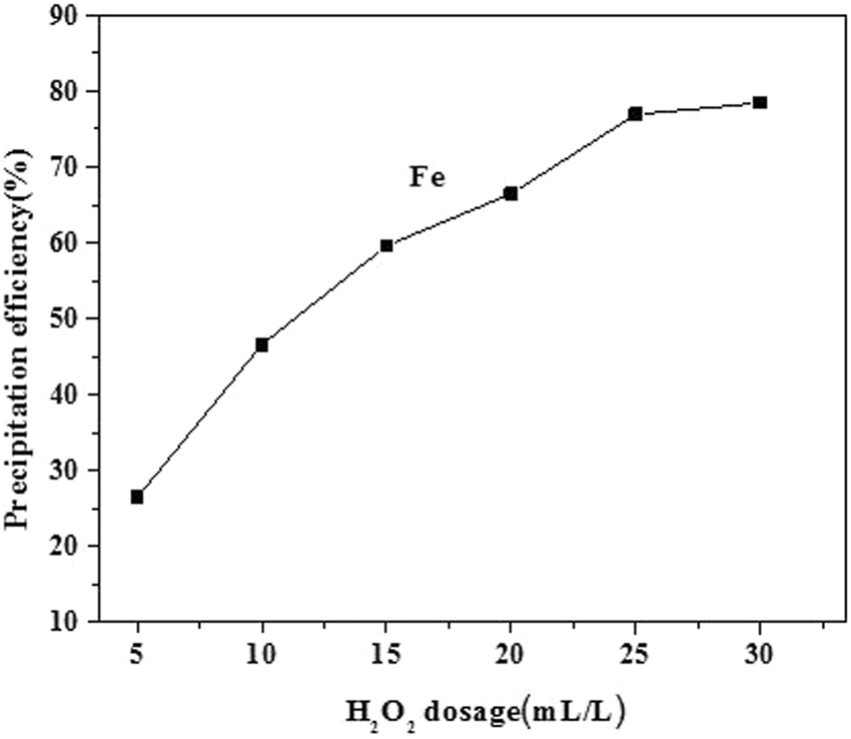
Effect of H_2_O_2_ dosage.

The effect of the stirring speed on the removal of Fe was investigated in the range of 200 rpm to 400 rpm, and the results are shown in Fig. 7. The removal of Fe increased with increasing stirring speed due to increased mass transport. However, high stirring speeds can also increase solvent evaporation, which can concentrate the solution and reduce the leaching efficiency, leading to loss of valuable metal. Thus, 300 rpm was identified as the optimal stirring speed. The effect of H_2_O_2_ dosage on the precipitation of iron was investigated in the range of 5–25 mL/L, as shown in Fig. 8. The Fe removal efficiency increased with increasing H_2_O_2_ dosage. This may be attributed to the fact that increasing H_2_O_2_ dosage improves the activity of reactants, resulting in a shift of the equilibria of Eq. (7) toward the right side, so the optimal H_2_O_2_ dosage is 25 mL/L.

### Concentration by evaporation and crystallization by cooling

Reducing the crystallization temperature can increase the first crystallization efficiency and promote grain growth. However, an excessive decrease in crystallization temperature not only increases the cost of refrigeration but also produces large quantities of needles and divergent crystals that reduce the zinc content of the product^23^. The effect of the crystallization temperature (10 °C to 35 °C) on the crystallinity of ZnSO_4_∙7H_2_O was investigated, and the results are shown in Table 7. At 20 °C, the first crystallization efficiency is relatively high, and the mass of the product is good. Although the first crystallization efficiency can be improved at a reduced temperature, the mass of the product would also be reduced. The mass loss of pure ZnSO_4_∙7H_2_O on heating is 43.90%, while at 20 °C, the mass loss of the product on heating is 43.40%. A value close to that of pure ZnSO_4_∙7H_2_O indicates that the quality of the product is excellent. Therefore, the optimal cooling temperature is 20 °C^24^.

**Table 7:** Effect of cooling temperature on ZnSO_4_∙7H_2_O quality.

Cooling temperature/°C	Crystal mass (g)	First Crystallization efficiency (%)	Mass loss on heating (%)
10	27.5	66.5	44.6
20	24.2	58.3	43.4
25	19.4	46.9	43.0
30	17.2	41.6	42.6
35	11.0	26.5	41.8

The effect of the crystallization time on the crystallinity of ZnSO_4_∙7H_2_O was investigated, and the results are shown in Table 8. At longer crystallization times, the first crystallization efficiency and the mass loss on heating continuously increased. This indicates that the crystallization time and efficiency of the formation changed with water content. When the crystallization time was 60 min, the first crystallization efficiency was higher, and the mass loss on heating was closer to the standard value (43.9%). Therefore, a crystallization time of 60 min was used throughout^23,24^.

**Table 8:** Effect of crystallization time on ZnSO_4_∙7H_2_O quality.

Crystallization time/°C	Crystal mass (g)	First Crystallization efficiency (%)	Mass loss on heating (%)
15	3.3	6.07	41.8
30	17.0	30.9	42.4
45	34.2	62.2	43.0
60	35.7	64.8	43.4
75	37.7	68.5	44.7

**Figure 9:**
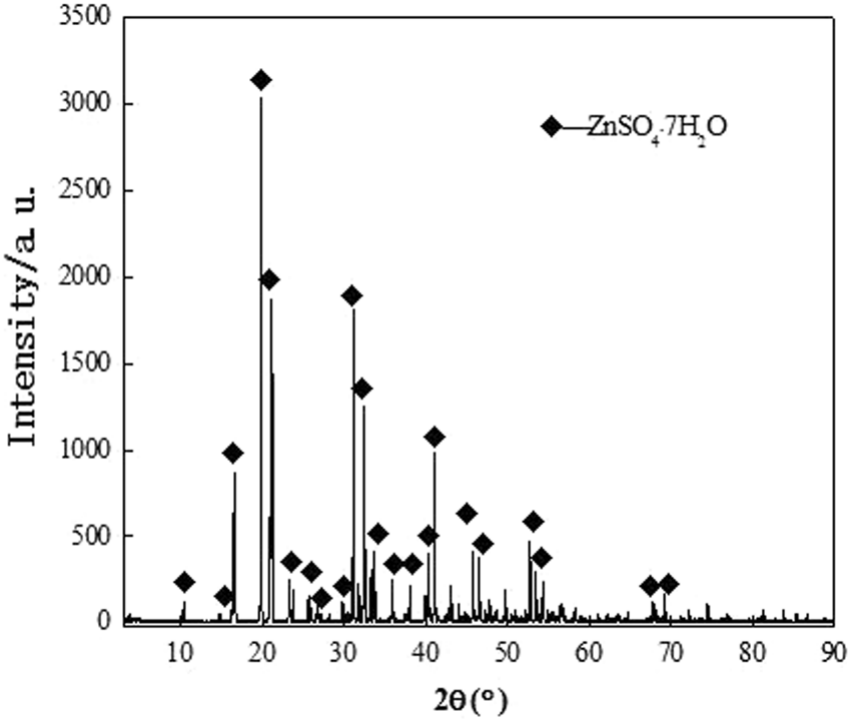
XRD pattern of ZnSO_4_∙7H_2_O.

**Figure 10:**
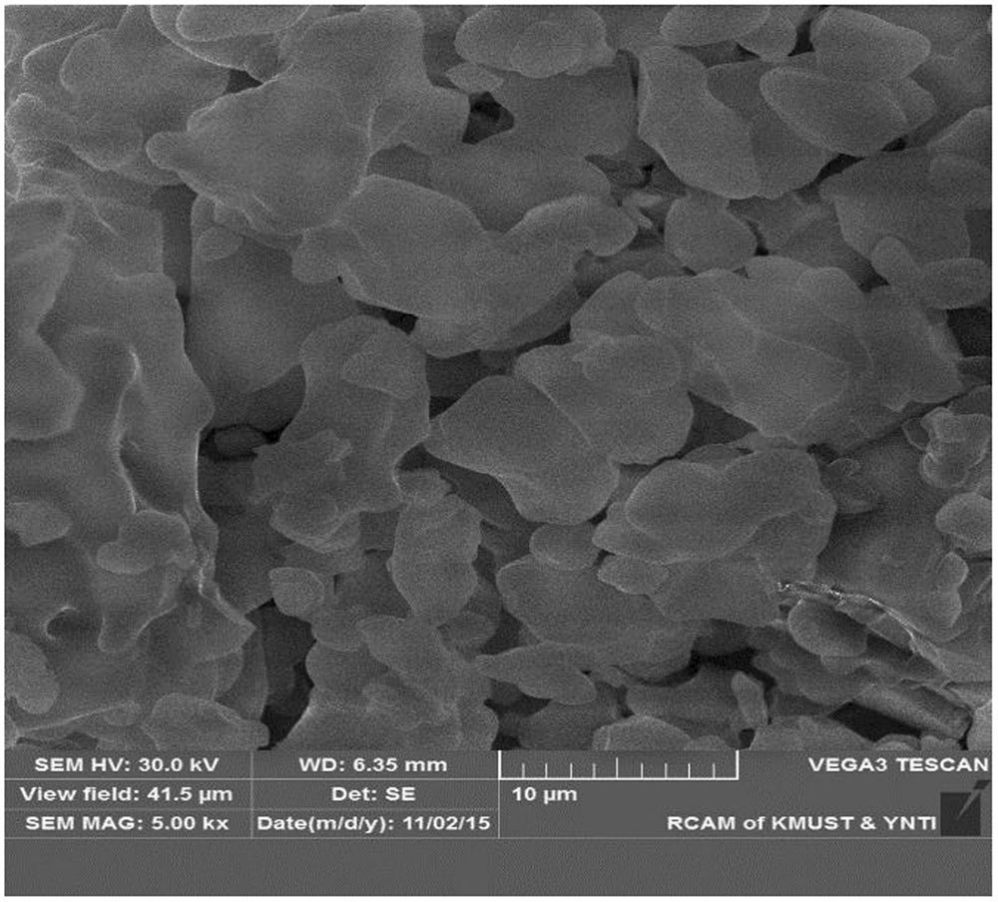
SEM image of ZnSO_4_∙7H_2_O.

**Figure 11:**
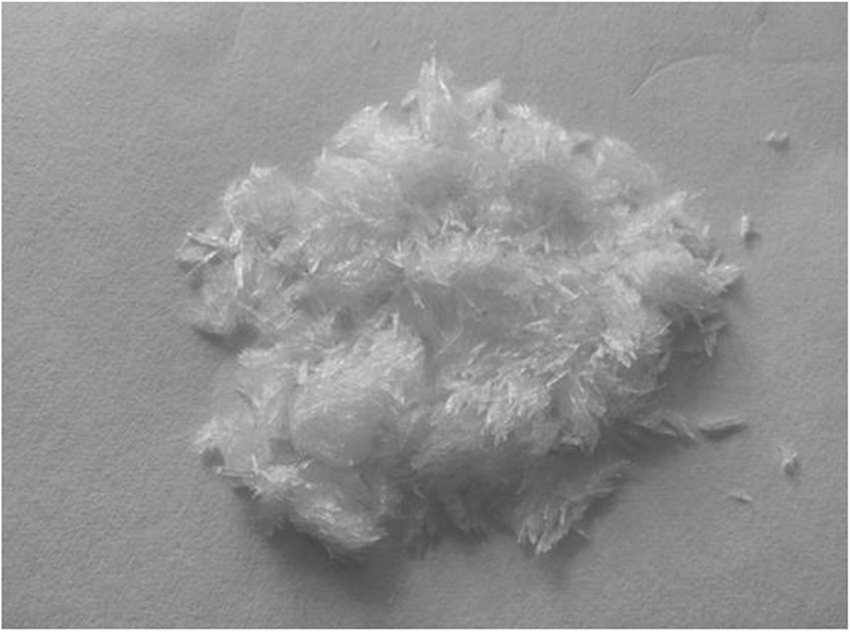
Physical figure of ZnSO_4_∙7H_2_O product.

Under the conditions of the optimal parameters, the content of ZnSO_4_∙7H_2_O in the product obtained by chemical analysis was 98.6%, and the zinc recovery efficiency was 97.5%. The contents of Ca, Mg, Fe, and Mn in the product were 0.178%, 0.028%, 0.023%, and 0.016%, respectively. The XRD and SEM patterns of the product were investigated, and the results of the analysis are shown in Figs 9 and 10, respectively. The chemical analysis and XRD analysis indicate that the product exists in the form of ZnSO_4_∙7H_2_O and has a high purity. The SEM shows granular structures, and the distribution of the laminated structure is quite homogeneous. The particle size is larger than that of the raw materials. Figure 11 shows that the experimentally obtained ZnSO_4_∙7H_2_O has an acicular surface structure.

## Conclusions

The circulative and accumulative problems of calcium and magnesium ions in the zinc process are successfully resolved by the preparation of ZnSO_4_·7H_2_O using filter cakes enriched in calcium and magnesium. The preparation process of ZnSO_4_·7H_2_O includes acid leaching, removal of impurities, concentration by evaporation and crystallization by cooling. The results of the present work show that the optimal leaching conditions are a solid to liquid ratio of 1:3.5, sulfuric acid concentration of 16%, leaching time of 20 min, final pH of 4.1–4.4, cooling and settling time of 120 min, oxidation time of 20 min, stirring speed of 300 rpm, H_2_O_2_ dosage of 25 mL/L, crystallization temperature of 20 °C, and crystallization time of 60 min. The zinc recovery efficiency is more than 95%, and the content of ZnSO_4_∙7H_2_O in the product is over 98%. This process is characterized by having a simple flow and a low cost.
